# Intestinal Immune Responses to Type 2 Oral Polio Vaccine (OPV) Challenge in Infants Previously Immunized With Bivalent OPV and Either High-Dose or Standard Inactivated Polio Vaccine

**DOI:** 10.1093/infdis/jix556

**Published:** 2018-01-03

**Authors:** Elizabeth B Brickley, Carolyn B Strauch, Wendy F Wieland-Alter, Ruth I Connor, Shu Lin, Joshua A Weiner, Margaret E Ackerman, Minetaro Arita, M Steven Oberste, William C Weldon, Xavier Sáez-Llorens, Ananda S Bandyopadhyay, Peter F Wright

**Affiliations:** 1 Department of Epidemiology, Geisel School of Medicine, Dartmouth College, Hanover; 2 Dartmouth College, Hanover; 3 Department of Pediatrics, Dartmouth-Hitchcock Medical Center, Lebanon; 4 Department of Microbiology and Immunology, Geisel School of Medicine, Dartmouth College, Hanover, New Hampshire; 5 Thayer School of Engineering, Dartmouth College, Hanover, New Hampshire; 6 Department of Virology II, National Institute of Infectious Diseases, Tokyo, Japan; 7 Division of Viral Diseases, Centers for Disease Control and Prevention, Atlanta, Georgia; 8 Hospital del Niño “Dr José Renán Esquivel” and Senacyt, Panama City, Panama; 9 Bill & Melinda Gates Foundation, Seattle, Washington

**Keywords:** poliovirus, inactivated vaccine, live oral vaccine, human challenge, mucosal immunity

## Abstract

**Background:**

The impact of inactivated polio vaccines (IPVs) on intestinal mucosal immune responses to live poliovirus is poorly understood.

**Methods:**

In a 2014 phase 2 clinical trial, Panamanian infants were immunized at 6, 10, and 14 weeks of age with bivalent oral polio vaccine (bOPV) and randomized to receive either a novel monovalent high-dose type 2–specific IPV (mIPV2HD) or a standard trivalent IPV at 14 weeks. Infants were challenged at 18 weeks with a monovalent type 2 oral polio vaccine (mOPV2). Infants’ intestinal immune responses during the 3 weeks following challenge were investigated by measuring poliovirus type-specific neutralization and immunoglobulin (Ig) A, IgA1, IgA2, IgD, IgG, and IgM antibodies in stool samples.

**Results:**

Despite mIPV2HD’s 4-fold higher type 2 polio D–antigen content and heightened serum neutralization profile, mIPV2HD-immunized infants’ intestinal immune responses to mOPV2 challenge were largely indistinguishable from those receiving standard IPV. Mucosal responses were tightly linked to evidence of active infection and, in the 79% of participants who shed virus, robust type 2–specific IgA responses and stool neutralization were observed by 2 weeks after challenge.

**Conclusions:**

Enhancing IPV-induced serum neutralization does not substantively improve intestinal mucosal immune responses or limit viral shedding on mOPV2 challenge.

**Clinical Trials Registration:**

NCT02111135.

## Teaser

These results from a 2014 phase 2 clinical trial in Panamanian infants suggest that high-dose inactivated polio vaccines (IPVs), like standard IPVs, are largely ineffective at inducing levels of intestinal immune responses requisite to control poliovirus shedding.

**(See the editorial commentary by Sutter, on pages 344–6.)**

The worldwide eradication of wild poliovirus type 2 was officially declared in September 2015 [1]. The signing of the declaration marked a major milestone for the Global Polio Eradication Initiative, which has reduced the incidence of polio by >99.99% and contributed to the prevention of an estimated 16 million cases of paralytic poliomyelitis since its launch in 1988 [2, 3]. Additionally, the wild-type 2 eradication achieved a key policy prerequisite specified in the Polio Eradication and Endgame Strategic Plan 2013–2018, thus helping to set into motion the globally coordinated serotype-by-serotype withdrawal of oral polio vaccines (OPVs) from routine immunization programs [4]. Between 17 April and 1 May 2016, >150 countries and territories successfully implemented the first phase of OPV cessation: a globally synchronized “switch” from the trivalent oral polio vaccine (tOPV), which targets poliovirus types 1, 2, and 3, to the bivalent oral polio vaccine (bOPV), which lacks a type 2 component.

Though tOPV has acted as a strategic lynchpin for eradication efforts in developing country settings to date, the replacement of OPVs with their inactivated polio vaccine (IPV) counterparts is now warranted to achieve and sustain polio eradication. After administration via oral drops, the live attenuated viruses that comprise OPVs replicate at vaccinees’ nasopharyngeal and gastrointestinal mucosal surfaces but, in very rare cases, accumulate genetic mutations that enable them to reacquire central nervous system virulence and/or heightened transmissibility (reviewed in [5]). The resulting circulating vaccine-derived polioviruses (cVDPVs) have caused >84% of the polio cases confirmed in 2017 (n = 80/95 year-to-date, as of 22 November 2017) [6] and continued to be identified through environmental surveillance (eg, detection of type 2 cVDPV in Nigeria in 2016 [7]). Moreover, the type 2 component of live oral polio vaccine (OPV2) is estimated to have caused between 100 and 200 annual cases of vaccine-associated paralytic poliomyelitis (VAPP) [8] during each of the 16 intervening years between the last known case of wild poliovirus type 2 and the tOPV-bOPV switch [9]. IPV, in contrast, confers substantial protection against paralytic poliomyelitis (ie, seroconverting approximately 80% of infants 10 weeks of age or older against all 3 types of polio after 2 doses [10]) and poses no associated risks of cVDPVs or VAPP.

Nevertheless, the ability of IPV immunization to generate a primary mucosal immune response with the capacity to inhibit live polio replication and thereby control poliovirus transmission remains uncertain (for an in-depth review, see [11]). Several studies of poliovirus-specific immunoglobulin (Ig) A have provided evidence that prior exposure to live poliovirus, via OPV or environmental exposure, appears to be important for inducing mucosal responses to IPV immunization [12, 13], and a 2012 systematic review has demonstrated that, when delivered in the absence of OPV, IPV fails to statistically significantly reduce children’s odds of having detectable fecal shedding following challenge with live attenuated polioviruses [14]. Emerging evidence from integrated bOPV/IPV trials, however, points to a potentially more significant role for IPV in the induction of mucosal immunity. For example, a 2013 trial of IPV-bOPV vaccine regimens in Chile reported significant inverse correlations between infants’ prechallenge poliovirus type 2–specific reciprocal serum neutralization titers and their fecal shedding indices (a composite metric accounting for both magnitude and duration of shedding) after mOPV2 challenge [15]. Moreover, in a 2013 Latin American trial in which a subset of infants was immunized with bOPV at 6, 10, and 14 weeks, vaccinees who received a supplementary dose of IPV at 14 weeks had modestly higher type 2–specific stool neutralization at mOPV2 challenge and lower viral shedding indices in the 4 weeks following challenge than their peers who received bOPV alone [16, 17]. While these studies suggest that, when delivered at standard doses as part of primary vaccine series, IPV may play a limited role in the induction of mucosal immunity, they also pose questions of whether it would be possible to enhance the mucosal immunogenicity of IPVs, such as via high-dose, adjuvanted, or intradermally administered vaccines, in order to mitigate shedding on subsequent exposure to live poliovirus.

To begin to address these questions, the current study investigated the effect of a novel monovalent high-dose type 2–specific IPV (mIPV2HD) on mucosal immunity in infants immunized during an observer-blind, comparative, randomized, phase 2 clinical trial in Panama [18]. In the trial, infants were immunized at 6, 10, and 14 weeks of age with bOPV and randomized 1:1 to receive a supplementary dose at 14 weeks of either mIPV2HD or standard trivalent IPV. All infants were then challenged with mOPV2 at 18 weeks of age and fecal samples were collected for the following 3 weeks [18]. For the principal trial results, Sáez-Llorens and colleagues reported that, in comparison to immunization with the standard IPV, mIPV2HD resulted in a higher proportion of infants with type 2 poliovirus seroconversion (ie, 75% in IPV vs 93% in mIPV2HD; *P* < .001) and no associated vaccine-related adverse events [18]. Nevertheless, 84% of infants in the trial had detectable mOPV2-related viral shedding despite having type 2–specific serum responses [18]. To examine intestinal mucosal immune responses to mOPV2 challenge in greater detail, stool samples collected during the trial were assayed for type-specific polio pseudovirus neutralization and for total and type-specific IgA, IgA1, IgA2, IgD, IgG, and IgM. Mucosal immune markers were compared by vaccine group assignment and by participants’ control of mOPV2-associated viral shedding.

## METHODS

### Study Design and Participants

The design of the phase 2, observer-blind, comparative, randomized controlled trial (NCT02111135), which enrolled infants at a single center in Panama City, Panama, between 14 April and 9 May 2014, has been described in detail previously [18]. Participants included healthy infants (restricted to 1 per household) aged 5–8 weeks at enrollment and excluded infants who (*i*) had been previously vaccinated against poliovirus; (*ii*) had a confirmed or suspected immunodeficiency; (*iii*) had a low birth weight (ie, <2500 g); (*iv*) had a known allergy to any component of the vaccines; or (*v*) resided in a household with someone who had received OPV within the previous 3 months or was scheduled to receive OPV during the study period. In the trial, all enrolled infants were vaccinated with 3 serial doses of bOPV (Sanofi Pasteur, Lyon, France) at approximately 6, 10, and 14 weeks of age and were randomized 1:1 to receive 1 additional intramuscular dose of either mIPV2HD formulated with 32 D-antigen units of poliovirus type 2 (Bilthoven Biologicals, Bilthoven, Netherlands) or standard IPV with 40, 8, and 32 D-antigen units of poliovirus types 1, 2, and 3, respectively (Sanofi Pasteur, Lyon, France) at the time of the third bOPV administration. All infants were challenged with a single dose of mOPV2 at 18 weeks of age. Serum samples were collected at 6, 14, 15, 18, and 19 weeks of age; stool samples were collected at 19, 20, and 21 weeks of age. See Supplementary Table 1 for study schema.

### Laboratory Procedures

Intestinal immunity against type 2 polio was evaluated from infant stool samples (5–10 g), which were shipped frozen first to the Polio and Picornavirus Laboratory Branch at the US Centers for Disease Control and Prevention (CDC) for the evaluation of viral shedding and subsequently to the Geisel School of Medicine at Dartmouth College for the current investigation. Poliovirus shedding is expressed as the log_10_ 50% cell culture infective dose (CCID_50_) per gram of stool, as described in the primary study [18]; samples with shedding below the limit of detection were recorded at a log_10_ CCID_50_ of zero. Vaccine group assignments were unblinded to the Dartmouth team only after sample testing and initial statistical analyses were completed. Mucosal immune responses were evaluated by methods described previously [17, 19, 20]. Stool neutralization titers were determined by limiting dilution inhibition of luciferase-labeled type-specific polio pseudoviruses in vitro as previously described [20] and expressed as the log_2_ reciprocal dilution needed to achieve 60% neutralization. Titers >1:512 (ie, the highest dilution tested) were recorded at 1:1024; those <1:4 (ie, the lowest dilution tested) were recorded at 1:2. Levels of IgA, IgA1, IgA2, IgD, IgG, and IgM in stool specimens were quantified relative to standard curves using a multiplex antibody platform as previously described [19] (see Supplementary Figure 1 for an overview of the method and Supplementary Table 2 for updated reagent manufacturing information). Relative concentration units below the limit of detection were recorded at a concentration of half the lowest detectable measurement of the given immunoglobulin. Serotype-specific serum neutralization activity was evaluated in the primary study at the CDC using the WHO standard microneutralization assay [21].

### Statistical Analyses

For the statistical analyses, infants were categorized both by vaccine group assignment (ie, bOPV-bOPV-bOPV+mIPV2HD or bOPV-bOPV-bOPV+IPV) and by shedding (ie, whether the infant had any or no viral shedding detectable during the postchallenge study visits). Differences in the distributions of log_10_ viral shedding, log_2_ serum and stool neutralization titers, and log_10_ mucosal immunoglobulin relative concentration units across groups were evaluated using the Mann–Whitney *U* test. Differences in the proportions of infants with detectable levels of log_10_ viral shedding, log_2_ serum and stool neutralization titers, and log_10_ mucosal immunoglobulin relative concentration units across groups were compared using Pearson χ^2^ test. Correlations between serum neutralization and viral shedding, neutralization, and IgA in stool were estimated using Spearman rank correlation coefficients and visualized by plotting the median and interquartile ranges of each of the variables vs the mean for each quintile of serum neutralization. Longitudinal patterns were investigated using scatter plots with local regression (LOESS) curves and 95% confidence intervals (CIs) fitted by shedding category; mean differences between groups were estimated at 1, 2, and 3 weeks postchallenge using linear regressions adjusted for vaccine group assignment. Pairwise correlations between mucosal antibody concentrations and neutralization titers in stool samples, as well as correlations between each of these intestinal immune markers and the magnitude of viral shedding (CCID_50_), were estimated after stratification by shedding group with Spearman rank correlation coefficients and visualized in matrices using the “corrplot” R package, version 0.77 [22]. All *P* values are from 2-sided statistical tests, and all analyses were performed using Stata software version 13.0 (StataCorp LP, College Station, Texas) and R software, version 3.2.5.

### Ethics

The study was approved by the Committee for the Protection of Human Subjects at Dartmouth College, Hanover, New Hampshire, and by the local ethical review board of the Hospital del Niño “Dr José Renán Esquivel,” Panama City, Panama, and was conducted in accordance with the Declaration of Helsinki, the International Conference on Harmonisation guideline for Good Clinical Practice, and the codes and regulations of Panama regarding research on human subjects. The initial consenting included provisions for the use of samples in future polio-related studies, and the reconsenting of participants was judged unnecessary.

## RESULTS

Intestinal immune responses to mOPV2 challenge were evaluated in 300 fecal samples collected from a randomly selected subset of 100 infants, representing approximately half of the participants in the primary study. The participants included 53 infants immunized with bOPV-bOPV-bOPV+mIPV2HD and 47 infants immunized with bOPV-bOPV-bOPV+IPV. In stool samples collected at 19 weeks of age, the mean total concentrations for the mucosal antibodies were 620000 ng/mL for IgA, 570 ng/mL for IgM, 27 ng/mL for IgD, and 1.7 ng/mL for IgG. Consistent with prior reports on duodenal secretions [23], poliovirus-specific IgG was not detected in any of the fecal samples. Although this study’s analyses focused on type 2–specific intestinal responses, it bears noting that infants in both vaccine groups exhibited robust mucosal immunity against poliovirus types 1 and 3, with high levels of type 1– and type 3–specific intestinal neutralization and IgA measured up to 7 weeks after the third dose of bOPV (Table 1).

**Table 1.** Polio Type-Specific Immune Profiles of Infants Previously Immunized With Bivalent Oral Polio Vaccine (bOPV)–bOPV-bOPV + Monovalent High-Dose Type 2–Specific Inactivated Polio Vaccine (n = 53) or bOPV-bOPV-bOPV + Trivalent Inactivated Polio Vaccine (n = 47)

		Weeks of Age	Weeks After Challenge	Median (IQR)		*P* Value, Mann–Whitney *U* Test	Proportion With Detectable Marker, No. (%)		*P* Value, χ^2^ Test
				bOPV-bOPV- bOPV+mIPV2HD	bOPV-bOPV- bOPV+IPV		bOPV-bOPV- bOPV+mIPV2HD	bOPV-bOPV- bOPV+IPV	
Type 2	Serum	6	…	4.5 (3.2–5.5)	3.5 (2.5–5.5)	.10	42 (79)	33 (70)	.30
	neutralization,	14	…	2.8 (2.5–3.8)	2.5 (2.5–3.2)	.18	31 (58)	21 (45)	.17
	log_2_ titer	15	…	8.5 (7.2–9.5)	5.2 (4.2–7.2)	<.001	52 (98)	46 (98)	.93
		18	…	7.5 (6.2–9.2)	5.8 (3.8–7.2)	<.001	53 (100)	43 (91)	.03
		19	1	8.5 (7.2–9.5)	7.5 (6.2–8.8)	.02	53 (100)	47 (100)	…
	Viral shedding,	19	1	5.0 (2.8–6.2)	4.0 (0–5.7)	.10	42 (79)	33 (70)	.30
	log_10_ CCID_50_	20	2	2.8 (0–4.8)	2.8 (0–4.1)	.54	31 (58)	28 (60)	.91
		21	3	0 (0–3.0)	0 (0–4.5)	.22	19 (36)	22 (47)	.27
	Stool neutralization,	19	1	6.3 (4.6–7.3)	5.6 (3.0–7.1)	.52	44 (83)	38 (81)	.78
	log_2_ titer	20	2	8.2 (7.2–10)	7.8 (6.3–8.7)	.08	52 (98)	44 (94)	.25
		21	3	8.4 (7.7–10)	8.0 (6.3–10)	.10	51 (96)	42 (89)	.18
	Stool IgA, log_10_ relative	19	1	0.9 (0.2–1.6)	0.7 (-0.2–1.4)	.44	50 (94)	44 (94)	.88
	concentration units	20	2	2.1 (1.5–2.5)	1.6 (1.0–2.1)	.05	53 (100)	47 (100)	…
		21	3	1.8 (1.2–2.8)	1.6 (0.4–2.5)	.35	53 (100)	47 (100)	…
Type 1	Stool neutralization,	19	1	7.2 (5.0–8.0)	7.4 (5.2–8.2)	.49	46 (87)	46 (98)	.04
	log_2_ titer	20	2	8.1 (7.2–8.6)	8.3 (7.1–10)	.53	52 (98)	46 (98)	.93
		21	3	8.2 (7.1–10)	7.9 (7.0–9.0)	.45	51 (96)	43 (91)	.32
	Stool IgA, log_10_ relative	19	1	–0.3 (–1.3 to 0.6)	0 (–1.3 to 0.7)	.27	37 (70)	35 (75)	.61
	concentration units	20	2	0.3 (–0.3 to 0.8)	0.3 (–0.3 to 0.8)	.74	49 (92)	43 (91)	.86
		21	3	0.3 (–0.5 to 0.8)	0.3 (–0.7 to 0.8)	.75	49 (92)	39 (83)	.15
Type 3	Stool neutralization,	19	1	6.9 (4.2–8.0)	6.5 (3.5–8.2)	.58	48 (91)	41 (87)	.60
	log_2_ titer	20	2	8.0 (6.3–10)	7.4 (4.8–8.7)	.23	50 (94)	44 (94)	.88
		21	3	8.1 (6.1–10)	6.9 (4.4–8.5)	.06	49 (92)	40 (85)	.24
	Stool IgA, log_10_ relative	19	1	1.6 (0.9–2.2)	1.8 (1.1–2.1)	.83	52 (98)	47 (100)	.34
	concentration units	20	2	1.9 (1.4–2.2)	1.7 (1.2–2.1)	.37	53 (100)	47 (100)	…
		21	3	1.8 (1.4–2.3)	1.8 (1.0–2.2)	.44	52 (98)	47 (100)	.34

Abbreviations: bOPV, bivalent oral polio vaccine; CCID_50_, 50% cell culture infectious dose; IgA, immunoglobulin A; IPV, trivalent inactivated polio vaccine; mIPV2HD, monovalent high-dose inactivated polio type 2 vaccine.

### Effects of mIPV2HD on Serum Neutralization and Mucosal Immune Responses to mOPV2 Challenge

**Figure 1.**
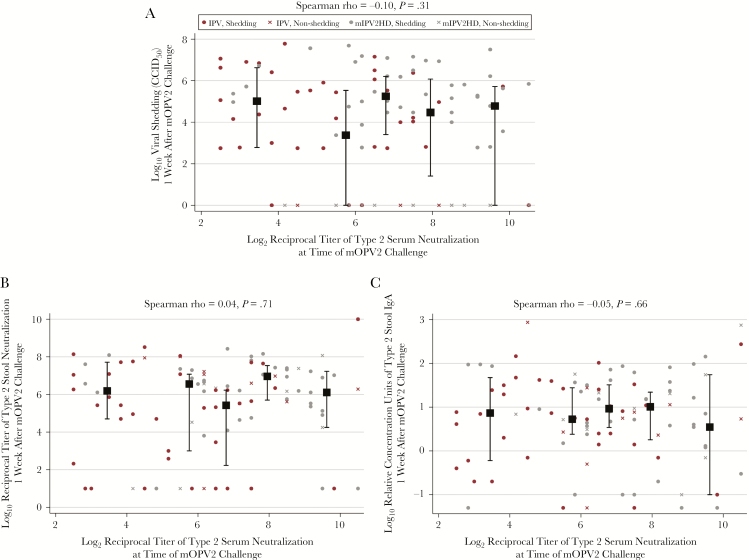
Correlations between polio type 2–specific neutralization titers measured in serum at the time of challenge and monovalent type 2 oral polio vaccine (mOPV2) viral shedding (50% cell culture infective dose [CCID_50_]) (*A*), polio pseudovirus type 2–specific neutralization titers (*B*), and polio type 2–specific immunoglobulin A (IgA) levels (*C*), all measured in stool at 1 week after mOPV2 challenge (ie, 19 weeks of age). Black squares and bars indicate the median and interquartile ranges of the variables on the y-axes within each quintile of serum neutralization plotted against the mean serum neutralization within each quintile. Red markers indicate infants in the bivalent oral polio vaccine (bOPV)–bOPV-bOPV + trivalent inactivated polio vaccine (IPV) group (n = 47); gray markers indicate infants in the bOPV-bOPV-bOPV + monovalent high-dose type 2–specific IPV (mIPV2HD) group (n = 53). Circle-shaped markers indicate infants with any detectable viral shedding during postchallenge study visits (n = 79); X-shaped markers indicate infants with no detectable viral shedding during postchallenge study visits (n = 21).

Although infants in the mIPV2HD group had significantly enhanced type 2 serum neutralization at the time of mOPV2 challenge compared with the IPV group (*P* < .001; Table 1), the markers of type 2–specific intestinal mucosal immunity measured in this investigation did not differ substantively by vaccine group assignment (Tables 1 and 2). As previously reported [18] and observed here, similar magnitudes of viral shedding occurred in both trial arms during the 3 follow-up visits (Table 1). Data collected on type 2–specific stool neutralization, IgA, IgA1, IgA2, IgD, and IgM similarly demonstrated comparable intestinal responses to mOPV2 challenge between the mIPV2HD and standard IPV groups in cross-sectional analyses at weeks 1, 2, and 3 postchallenge (Tables 1 and 2). In analyses that combined vaccine groups, type 2–specific serum neutralization titers were not associated with fecal levels of type 2–specific viral shedding, stool neutralization, nor IgA (Figure 1).

**Table 2.** Presence of Polio Type 2–Specific Mucosal Antibodies Measured in Stool Samples Collected From Infants During Postchallenge Study Visits

	Total No.	Proportion With Detectable Type 2–Specific Antibody									
		IgA, No. (%)	*P* Value, χ^2^ Test	IgA1, No. (%)	*P* Value, χ^2^ Test	IgA2, No. (%)	*P* Value, χ^2^ Test	IgD, No. (%)	*P* Value, χ^2^ Test	IgM, No. (%)	*P* Value, χ^2^ Test
1 week postchallenge (19 wk of age)											
Vaccine group			.88		.895		.748		.520		.571
bOPV-bOPV- bOPV+mIPV2HD	53	50 (94%)		13 (25%)		3 (6%)		34 (64%)		11 (21%)	
bOPV-bOPV- bOPV+IPV	47	44 (94%)		11 (23%)		2 (4%)		33 (70%)		12 (26%)	
Shedding during follow-up			.79		.982		.285		.280		.099
Shedding	79	74 (94%)		19 (24%)		3 (4%)		55 (70%)		21 (27%)	
Nonshedding	21	20 (95%)		5 (24%)		2 (10%)		12 (57%)		2 (10%)	
2 weeks postchallenge (20 wk of age)											
Vaccine group			…		.129		.597		.571		.075
bOPV-bOPV- bOPV+mIPV2HD	53	53 (100%)		26 (49%)		10 (19%)		42 (79%)		25 (47%)	
bOPV-bOPV- bOPV+IPV	47	47 (100%)		16 (34%)		7 (15%)		35 (74%)		14 (30%)	
Shedding during follow-up			…		.004		.093		.206		.009
Shedding	79	79 (100%)		39 (49%)		16 (20%)		63 (80%)		36 (46%)	
Nonshedding	21	21 (100%)		3 (14%)		1 (5%)		14 (67%)		3 (14%)	
3 weeks postchallenge (21 wk of age)											
Vaccine group			…		.594		.416		.124		.631
bOPV-bOPV- bOPV+mIPV2HD	53	53 (100%)		22 (42%)		12 (23%)		45 (85%)		17 (32%)	
bOPV-bOPV- bOPV+IPV	47	47 (100%)		22 (47%)		14 (30%)		34 (72%)		13 (28%)	
Shedding during follow-up			…		<.001		.013		.338		.021
Shedding	79	79 (100%)		43 (54%)		25 (32%)		64 (81%)		28 (35%)	
Nonshedding	21	21 (100%)		1 (5%)		1 (5%)		15 (71%)		2 (10%)	

Abbreviations: bOPV, bivalent oral polio vaccine; Ig, immunoglobulin; IPV, trivalent inactivated polio vaccine; mIPV2HD, monovalent high-dose inactivated polio type 2 vaccine.

### Associations of mOPV2 Shedding With Serum Neutralization and Mucosal Immune Markers

**Figure 2.**
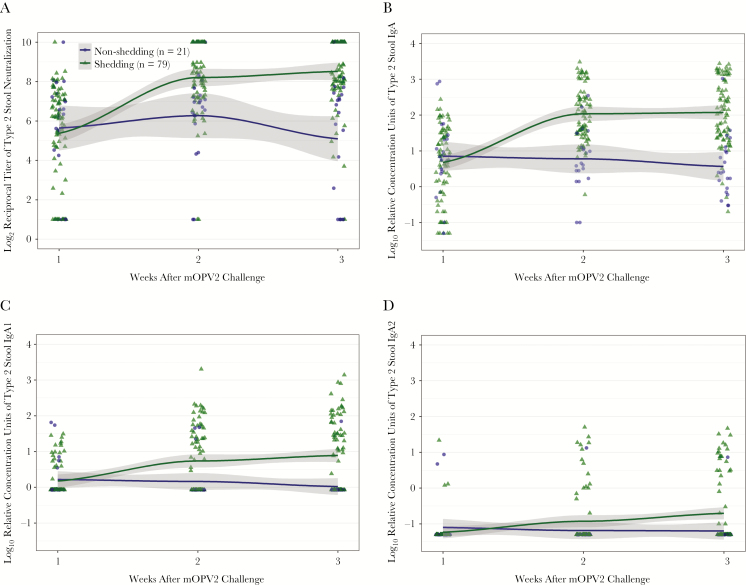
Polio type 2–specific intestinal immune responses to monovalent type 2 oral polio vaccine (mOPV2) challenge in infants with any (n = 79) and no (n = 21) viral shedding detectable during postchallenge study visits: stool neutralization titers (*A*), immunoglobulin (Ig) A levels (*B*), IgA1 levels (*C*), and IgA2 levels (*D*), all measured in stool at 1–3 weeks after mOPV2 challenge (ie, 19–21 weeks of age). Data represent the combined responses of both vaccine groups. Scatter plots indicate individual measurements. LOESS curves (95% confidence interval) were fitted by shedding category. Abbreviations: Ig, immunoglobulin.

Approximately one-fifth of infants (n = 11/53 in the mIPV2HD group; n = 10/47 in the IPV group) shed no detectable mOPV2 virus during the postchallenge study visits. Relative to the nonshedders, the infants who would go on to shed virus exhibited modestly lower mean type 2–specific serum neutralization activity during 4 of the study visits prior to challenge and significantly higher neutralization at the visit 1 week after challenge (*P* = .01; Table 3). Shedding status was also associated with the kinetics of the intestinal mucosal immune response to mOPV2 challenge. Whereas shedders (n = 79) and nonshedders (n = 21) had similar levels of type 2–specific stool neutralization and mucosal antibodies 1 week after challenge, the groups markedly diverged by 2 and 3 weeks after challenge (Figure 2; Tables 2 and 3). The shedding group had statistically significantly higher mean levels of type 2–specific stool neutralization activity, IgA, and IgA1 during weeks 2 and 3 postchallenge (Figure 2), and the differences in mucosal responses by shedding status were consistent regardless of IPV group assignment (Table 3; Supplementary Figure 2). In addition, relative to nonshedding infants, a larger proportion of shedding infants had detectable type 2–specific IgA1, IgA2, and IgM responses at 3 weeks after challenge (Table 2). Type 2–specific IgD was detectable in the majority of participants, but no significant differences by shedding status were observed for this parameter across any of the time nodes (Table 2).

**Table 3.** Polio Type 2–Specific Immune Responses to Monovalent Type 2 Oral Polio Vaccine Challenge in Infants With Any (n = 79) and No (n = 21) Viral Shedding Detectable During Postchallenge Study Visits

Type 2–Specific Immune Markers	Weeks of Age	Weeks After Challenge	Mean Difference by Shedding Status (95% CI) After Adjustment for Vaccine Group	*P* Value, Wald Test
Serum neutralization, log_2_ titer	6	…	–0.78 (–1.59 to .03)	.06
	14	…	–0.50 (–1.15 to .14)	.12
	15	…	–0.46 (–1.43 to .51)	.35
	18	…	–0.89 (–1.87 to .09)	.08
	19	1	1.11 (.27–1.94)	.01
Stool neutralization, log_2_ titer	19	1	–0.27 (–1.49 to .94)	.66
	20	2	1.93 (1.03–2.83)	<.001
	21	3	3.41 (2.43–4.39)	<.001
Stool IgA, log_10_ relative concentration units	19	1	–0.17 (–.67 to .33)	.50
	20	2	1.26 (.88–1.63)	<.001
	21	3	1.50 (1.06–1.95)	<.001
Stool IgA1, log_10_ relative concentration units	19	1	–0.04 (–.28 to .20)	.75
	20	2	0.57 (.15–.99)	.008
	21	3	0.87 (.43–1.32)	<.001
Stool IgA2, log_10_ relative concentration units	19	1	–0.13 (–.35 to .08)	.22
	20	2	0.26 (–.11 to .63)	.17
	21	3	0.49 (.06–.93)	.03
Stool IgD, log_10_ relative concentration units	19	1	0.13 (–.06 to .31)	.17
	20	2	0.11 (–.05 to .28)	.18
	21	3	0.07 (–.09 to .23)	.41
Stool IgM, log_10_ relative concentration units	19	1	0.16 (–.01 to .32)	.06
	20	2	0.29 (.09–.50)	.005
	21	3	0.24 (.04–.43)	.02

Mean differences comparing the shedders to the nonshedders (reference group) were estimated for each time node using linear regressions with adjustment for vaccine group assignment.

Abbreviations: CI, confidence interval; Ig, immunoglobulin.

### Correlates of Mucosal Immunity to Type 2 Poliovirus

**Figure 3.**
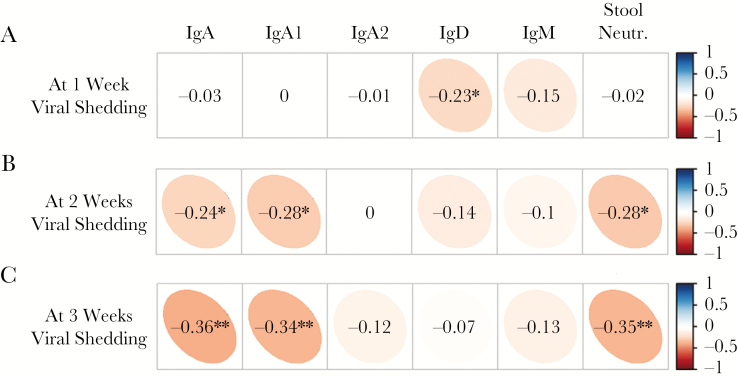
Correlations between levels of monovalent type 2 oral polio vaccine (mOPV2) viral shedding (50% cell culture infectious dose) and levels of polio type 2–specific mucosal antibodies and neutralization titers measured in stool collected at 1 week (*A*), 2 weeks (*B*), and 3 weeks (*C*) after mOPV2 challenge from infants with any viral shedding detectable during postchallenge study visits (n = 79). Spearman rank correlation coefficients were estimated from the combined responses of both vaccine groups. The narrowness of the ellipse and intensity of the color indicate the strength of a given correlation coefficient. The corresponding numerical values are defined by the vertical bar on the right. *P < .05; **P < .005. Abbreviations: CCID_50_, 50% cell culture infectious dose, Ig, immunoglobulin; Neutr., neutralization.

With the aim of exploring how intestinal antibody concentrations related to each other and to neutralizing activity over time, we examined pairwise correlations in the 2 shedding groups at 1, 2, and 3 weeks postchallenge (Supplementary Figure 3). Overall, type 2–specific IgA was the strongest correlate of stool neutralization (Spearman ρ at 2 weeks: 0.74, *P* < .0001) and explained approximately half of its variance in a univariate linear regression (*r*^*2*^ at 2 weeks: 0.47). Focusing on only the 79 infants in the shedding group, a pattern between the levels of immune markers and the magnitude of viral shedding emerged. At 1 week after challenge, there was no association between viral shedding and type 2–specific serum neutralization (Spearman ρ: 0.00, *P* = .99; see Supplementary Figure 4 for detailed scatter plot). Out of the intestinal immune markers, only type 2–specific IgD was statistically significantly inversely associated with viral shedding at 1 week (Spearman ρ: –0.23, *P* = .04; Figure 3). By weeks 2 and 3 after challenge, the inverse correlation between viral shedding and type 2–specific IgD waned as the inverse correlations between shedding and type 2–specific IgA, IgA1, and stool neutralization titers became stronger (*P* < .005 for all).

## DISCUSSION

In infants previously immunized with 3 doses of bOPV plus either mIPV2HD or standard IPV, the current study delineates intestinal immune response to live oral polio vaccine challenge using state-of-the-art multiplex bead–based tools for quantitating antibodies and polio pseudovirus neutralization assays for assessing function. The results of this analysis provide compelling evidence that enhancing serum neutralization with a high-dose IPV in the primary immunization series does not substantively improve mucosal protection upon subsequent exposure to live polioviruses. Despite mIPV2HD’s 4-fold higher concentration of type 2 polio antigen and 3-fold higher postvaccination median serum antibody titers, mIPV2HD-immunized infants’ intestinal immune responses to challenge did not differ from those of the standard IPV group in terms of polio type 2–specific IgA, IgA1, IgA2, IgD, IgG, or IgM antibody responses or neutralization at any time during the 3 weeks after mOPV2 challenge.

Overall, the current findings are consistent with earlier studies demonstrating a limited role for IPV—and specifically enhanced potency IPV [24]—in the induction of mucosal antibodies and inhibition of poliovirus shedding in individuals with no previous exposure to live virus [12–18, 23, 25]. For example, a 1991 trial in a Maryland cohort showed that, despite having significantly higher mean prechallenge serum neutralizing antibody levels, children immunized with 3 doses of enhanced potency IPV at 2, 4, and 18 months of age were significantly more likely to shed virus upon mOPV1 challenge than their peers who received OPV at the same time points (percentage shedding: 63% vs 25%) [24]. Moreover, a 2001 trial in a Sabin virus–free Cuban population found that the percentage of infants who shed any type of poliovirus after tOPV challenge was >90% regardless of whether the infants had received zero, 2, or 3 prior doses of IPV [25]. Moving forward, further research will be valuable for understanding whether administering standard or high-dose IPVs at later ages and/or with repeated doses could overcome these limitations and have the potential to augment the mucosal immunogenicity of inactivated vaccines.

Because the inactivated vaccines had a limited impact on type 2 viral shedding after challenge, this study provided a unique opportunity to explore the kinetics of the mucosal antibody response to live poliovirus and to gain novel insight into the correlates of intestinal immunity against polio. In line with prior investigations of intestinal immunity [17, 19, 23], these data suggest that IgA, and the IgA1 subclass in particular (as previously noted in serum responses to poliovirus in naive individuals [26]), is likely the primary mediator of intestinal neutralization of polio pseudoviruses. Specifically, IgA, IgA1, and intestinal neutralization responded by 2 weeks after challenge in both IPV groups, and marker levels were inversely correlated with the amount of virus recovered from the 79% of children with detectable shedding. In infants with no detectable viral shedding, levels of IgA, IgA1, and neutralization remained flat across the 3 postchallenge study visits. Intriguingly, low levels of poliovirus-specific IgD were widely detectable in vaccinated infants and appeared to be inversely correlated with shedding 1 week postchallenge, suggesting a potential involvement of IgD in immune surveillance and/or the early neutralizing response (for a broader overview of IgD and mucosal immunity, see [27]). Type 2–specific IgA2 and IgM became detectable in a substantial proportion of individuals with active infection, whereas type 2–specific IgG was detectable in no fecal samples.

While the present study described the longitudinal mucosal antibody response to oral polio vaccine challenge with unprecedented detail, the chief limitation was that stool samples were only collected in the original trial from infants during the 3 weeks following mOPV2 challenge. Without fecal samples collected from infants at the time of and in the weeks immediately following IPV immunization, this study was unable to fully assess and compare the mIPV2HD and IPV vaccines’ primary intestinal immunogenicity (ie, measured in terms of stool neutralization and antibody levels in lieu of viral shedding). Moreover, without fecal samples collected prior to infants’ first exposure to live attenuated type 2 poliovirus (ie, at the time of mOPV2 challenge), the study was unable to evaluate the change in infants’ immune parameters relative to pre-OPV levels or to compare indicators of baseline intestinal immunity between shedders and nonshedders. The second key limitation was that the original trial did not include a bOPV-only control group and, thus, obviated investigation of whether the mIPV2HD and IPV groups primed type 2 mucosal responses to live virus relative to children with no prior polio type 2 immunological experience. A third limitation is that tOPV was still in use for Panamanian routine immunization in 2014, and it remains plausible that the relatively large nonshedding group (21%) may have been influenced by inadvertent exposure to OPV-derived strains.

In conclusion, to prepare for the transitional and post-OPV phase of the polio endgame, it is important to appraise candidate immunization strategies in terms of their potential to both prevent paralytic polio and interrupt transmission of viruses. The findings of the current study reinforce the concept that, although vaccines such as mIPV2HD that induce strong serum neutralization are likely to be highly valuable for minimizing paralytic poliomyelitis risks [18], enhanced potency inactivated vaccines, on their own, are not likely to be effective at inducing the intestinal immune responses that are necessary to control poliovirus shedding and curtail outbreaks.

## Supplementary Data

Supplementary materials are available at *The Journal of Infectious Diseases* online. Consisting of data provided by the authors to benefit the reader, the posted materials are not copyedited and are the sole responsibility of the authors, so questions or comments should be addressed to the corresponding author.

## Supplementary Material

Supplementary Information

## Notes

***Author contributions.*** All authors had full access to the data in the study and share final responsibility for the decision to submit for publication.

***Disclaimer.*** The funder had no role in data collection.

***Financial support.*** This work was supported by the Bill & Melinda Gates Foundation (grant number OPP1104756 to P. F. W.).

***Potential conflicts of interest.*** A. S. B. is an employee of the study funder and was involved in study design, data interpretation, and writing of the report. All other authors report no potential conflicts of interest. All authors have submitted the ICMJE Form for Disclosure of Potential Conflicts of Interest. Conflicts that the editors consider relevant to the content of the manuscript have been disclosed.
