# Supplementary material for: Intestinal Immune Responses to Type 2 Oral Polio Vaccine (OPV) Challenge in Infants Previously Immunized With Bivalent OPV and Either High-Dose or Standard Inactivated Polio Vaccine
Source: J Infect Dis. 2018 Jan 3;217(3):371–80. doi: 10.1093/infdis/jix556 (PMC5853416; doi:10.1093/infdis/jix556)
Supplement: Supplementary Information [file jix556_suppl_supplementary_information.docx]

**Supplementary Information**

Supplementary Table 1. Simplified study schema for the ‘Safety study of a single dose of monovalent high-dose inactivated poliovirus type 2 vaccine (m-IPV2 HD) in infants in early life’ (NCT 02111135) in Panama City, Panama, between 14 April and 9 May 2014.

|  | Before Challenge | | | | | After Challenge | | |
| --- | --- | --- | --- | --- | --- | --- | --- | --- |
|  | **6 weeks**  **of age** | **10 weeks**  **of age** | **14 weeks**  **of age** | **15 weeks**  **of age** | **18 weeks**  **of age** | **19 weeks**  **of age** | **20 weeks**  **of age** | **21 weeks**  **of age** |
| All participants | 1^st^ serum sample |  | 2^nd^ serum sample | 3^rd^ serum sample | 4^th^ serum sample | 5^th^ serum sample |  |  |
|  |  |  |  |  |  | 1^st^ stool sample | 2^nd^ stool sample | 3^rd^ stool sample |
| mIPV2HD group | 1^st^ bOPV dose | 2^nd^ bOPV dose | 3^rd^ bOPV dose  + mIPV2HD dose |  | mOPV2 challenge |  |  |  |
| IPV group | 1^st^ bOPV dose | 2^nd^ bOPV dose | 3^rd^ bOPV dose  + IPV dose |  | mOPV2 challenge |  |  |  |

Abbreviations: bOPV = bivalent oral polio vaccine; mIPV2HD = monovalent high dose inactivated polio type 2 vaccine; IPV = trivalent inactivated polio vaccine.

Supplementary Table 2. Product information for the reagents used in the multiplex bead-based immunoassay.

|  | Target | Product Name | Supplier | Location | Type |
| --- | --- | --- | --- | --- | --- |
| On Bead | IgA | Anti-human IgA mAb | Mabtech, Inc. | Cincinatti, OH, USA | Clone MT57 |
|  | IgD | Goat anti-human IgD antibody | Bethyl Laboratories | Montgomery, TX, USA | Polyclonal |
|  | IgM | Anti-human IgM Antibody | AB Biotechnologies | Bloomington, IN, USA | Monoclonal |
|  | IgG | Goat Anti-Human IgG Fc | Southern Biotech | Birmingham, AL, USA | Polyclonal |
| Detection | IgA | Goat Anti-Human IgA-PE | Southern Biotech | Birmingham, AL, USA | Polyclonal |
|  | IgA1 | Anti-Human IgA1 antibody* | Abcam | Cambridge, UK | Clone RM124 |
|  | IgA2 | Anti-IgA2 antibody * | Abcam | Cambridge, UK | Clone RM125 |
|  | IgM | Mouse Anti-Human IgM-PE | Southern Biotech | Birmingham, AL, USA | Clone SA-DA4 |
|  | IgD | Mouse Anti-Human IgD delta chain (Phycoerythrin) | Abcam | Cambridge, UK | Clone IADB6 |
|  | IgG | Mouse Anti-Human IgG Fc-PE | Southern Biotech | Birmingham, AL, USA | Clone JDC-10 |
|  | Rabbit IgG | Goat anti-rabbit IgG-PE | Southern Biotech | Birmingham, AL, USA | Polyclonal |

* IgA1 and IgA2 antibodies were not PE labeled and required an additional incubation with anti-rabbit IgG-PE. Abbreviations: Ig = immunoglobulin; mAB = monoclonal antibody; FC = fragment crystallizable; PE = Phycoerythrin.

**Supplementary Figure 1. Schematic overview of the multiplexed assay to characterize immunoglobulins present in stool samples**. (A) First, whole inactivated poliovirus particles and isotyping reagents were covalently conjugated to fluorescently coded microspheres. (B) Second, to allow antibody isotypes and virus-specific antibodies to be affinity purified from the bulk mixture, conjugated microspheres were mixed with stool samples, and the bead-bound antibodies were detected with fluorescent secondary reagents specific for IgA, IgA1, IgA2, IgD, IgM, and IgG. (C) Finally, Mean Fluorescent signal Intensities (MFI) of the polio type-specific immunoglobulins were compared to those of an antibody standard to quantify relative concentrations. Measurements were considered to be detectable if the relative concentration units were greater than three standard deviations above the background level of control samples.

**
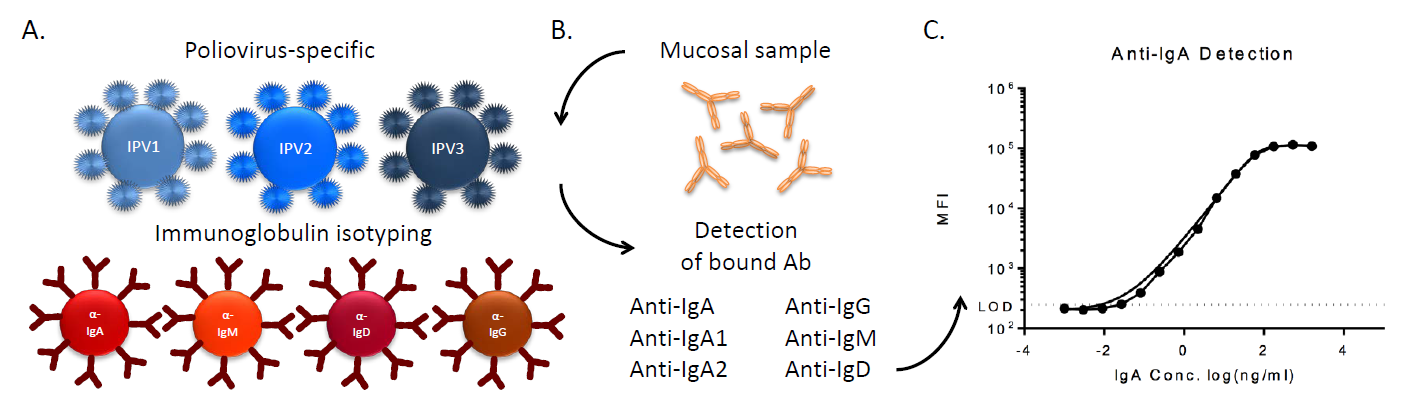
**

**Supplementary Figure 2. Polio type 2-specific intestinal immune responses to mOPV2 challenge by vaccine group assignment and shedding category: (A) neutralization titers, (B) IgA levels, (C) IgA1 levels, (D) IgA2 levels, (E) IgD levels, and (F) IgM levels, all measured in stool at one to three weeks after mOPV2 challenge (i.e., 19 to 21 weeks of age).** Scatter plots indicate individual measurements. LOESS curves (95% CI) were fitted by vaccine group and shedding category. Abbreviations: IPV = trivalent inactivated polio vaccine; mIPV2HD = monovalent high dose inactivated polio type 2 vaccine.

A.
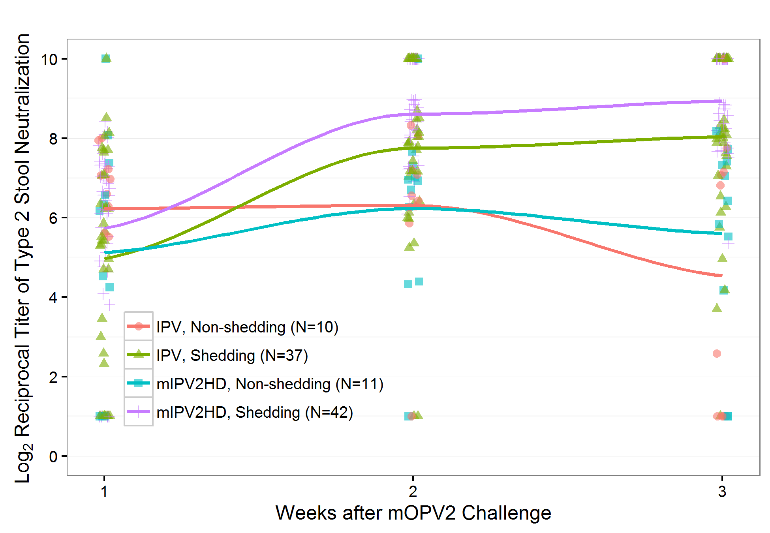
 B.
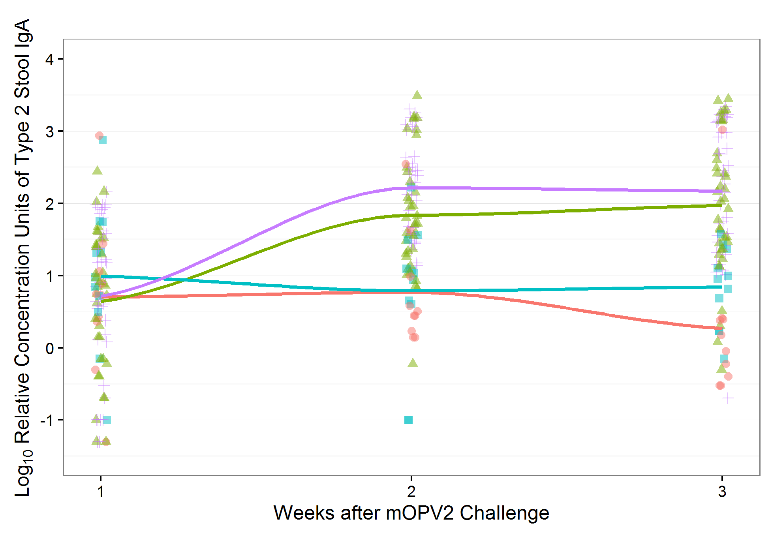


C.
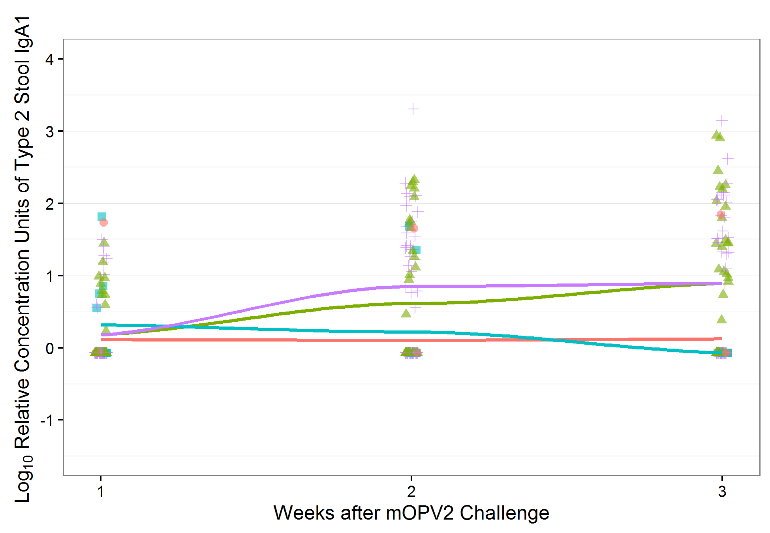
 D.
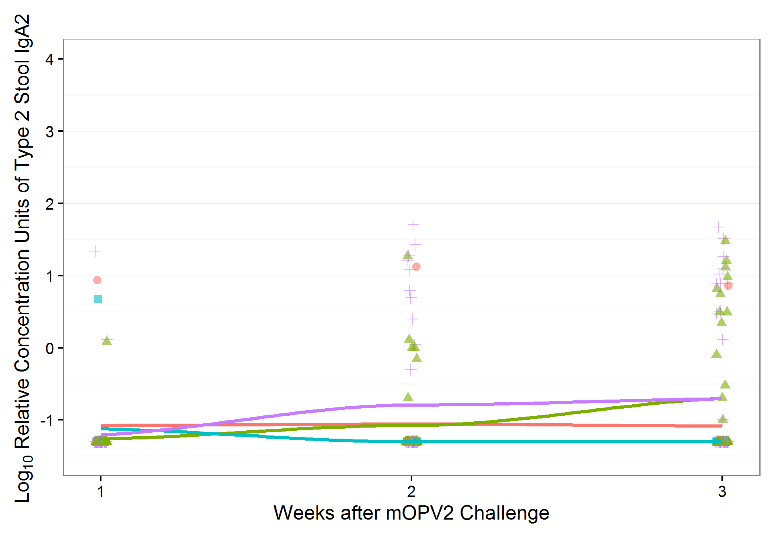


E.
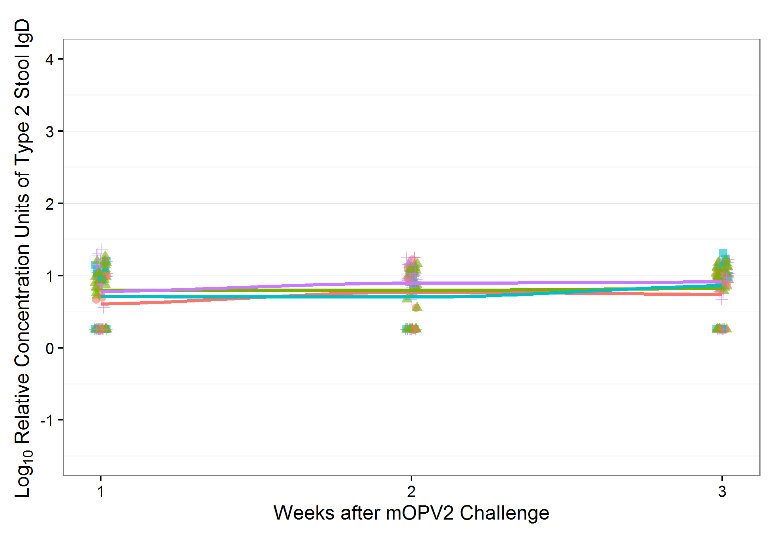
 F.**
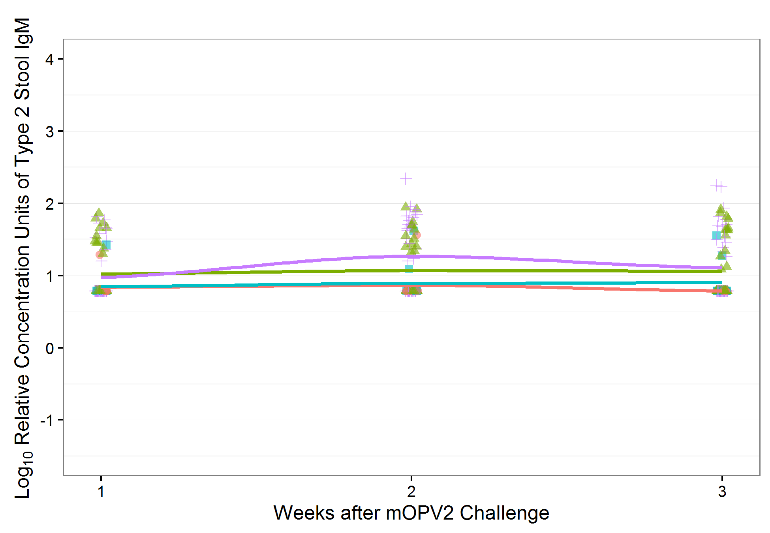
**

**Supplementary Figure 3. Pairwise correlations between polio type 2-specific mucosal antibodies and neutralization titers measured in stool collected at (A) one, (B) two, and (C) three weeks after mOPV2 challenge from infants with any (N=79) and no (N=21) viral shedding detectable during post-challenge study visits.** Spearman’s rank correlation coefficients were estimated from the combined responses of both vaccine groups. The narrowness of the ellipse and intensity of the color indicate the strength of a given correlation coefficient. Diagonal lines indicate perfect correlations. The corresponding numerical values are defined by the vertical bar on the right. Abbreviations: Ig, immunoglobulin; Neutr., neutralization.

|  | | **Shedding (N=79)** | **Non-shedding (N=21)** |
| --- | --- | --- | --- |
| **Time after mOPV2 Challenge** | **A. One Week** | 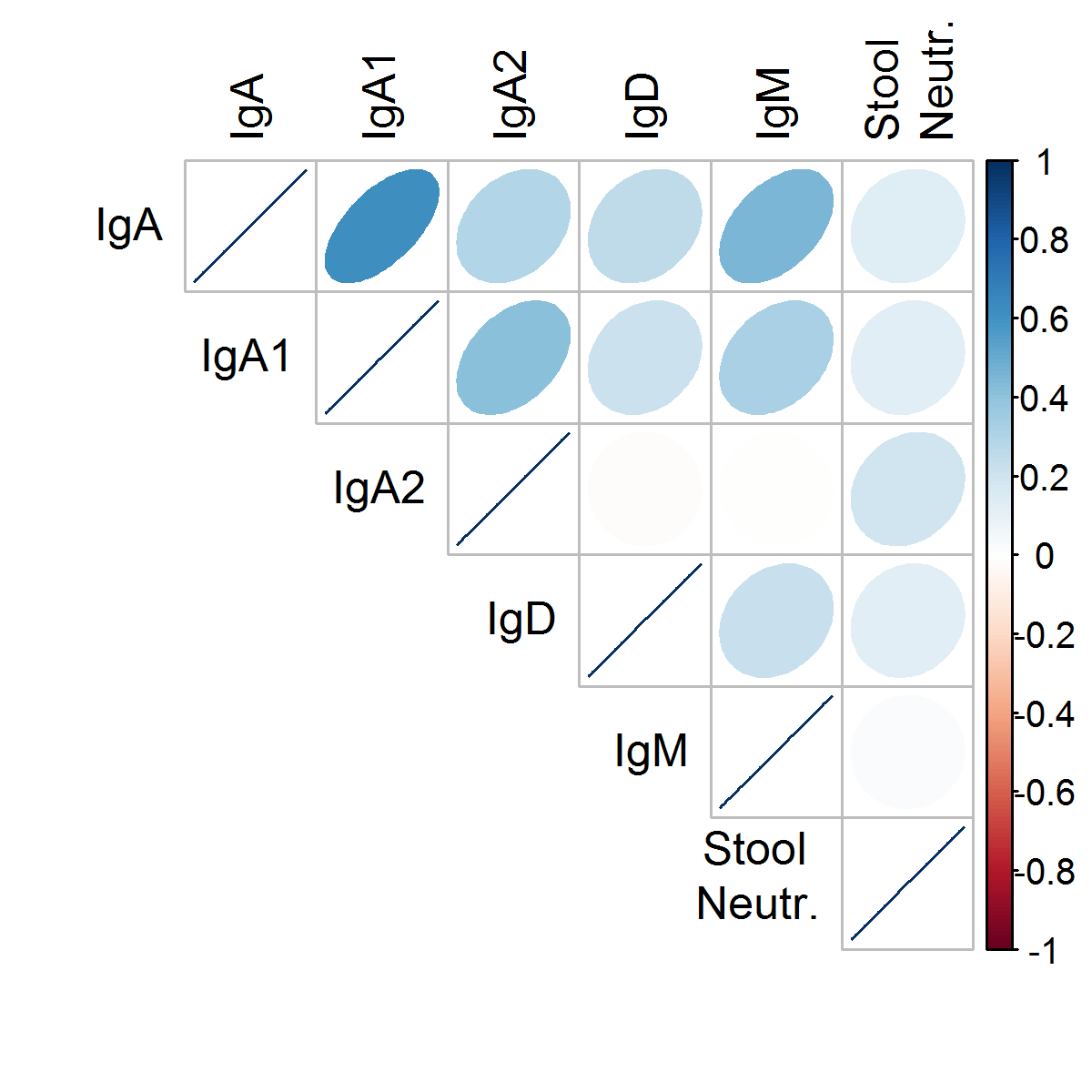 | 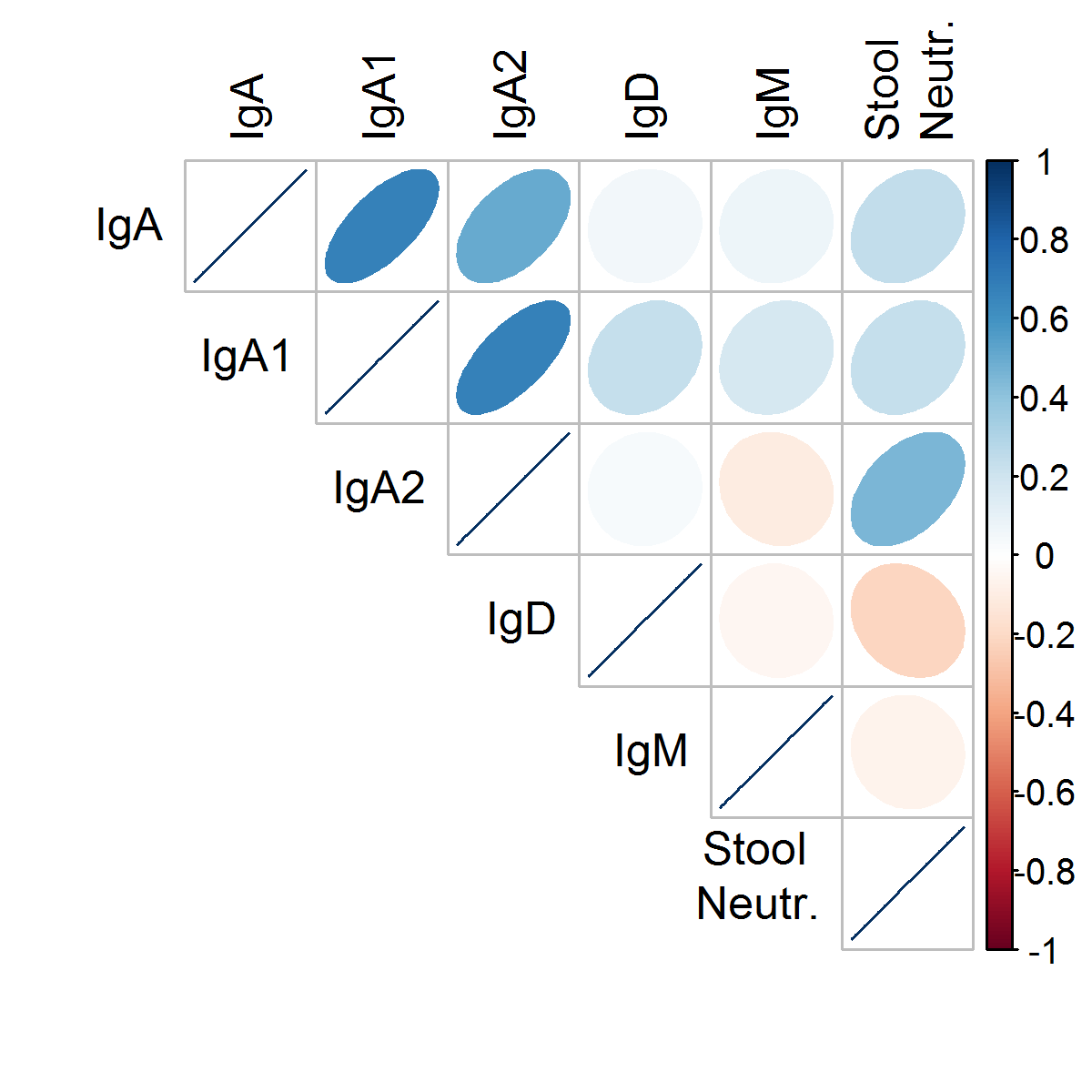 |
|  | **B. Two Weeks** | 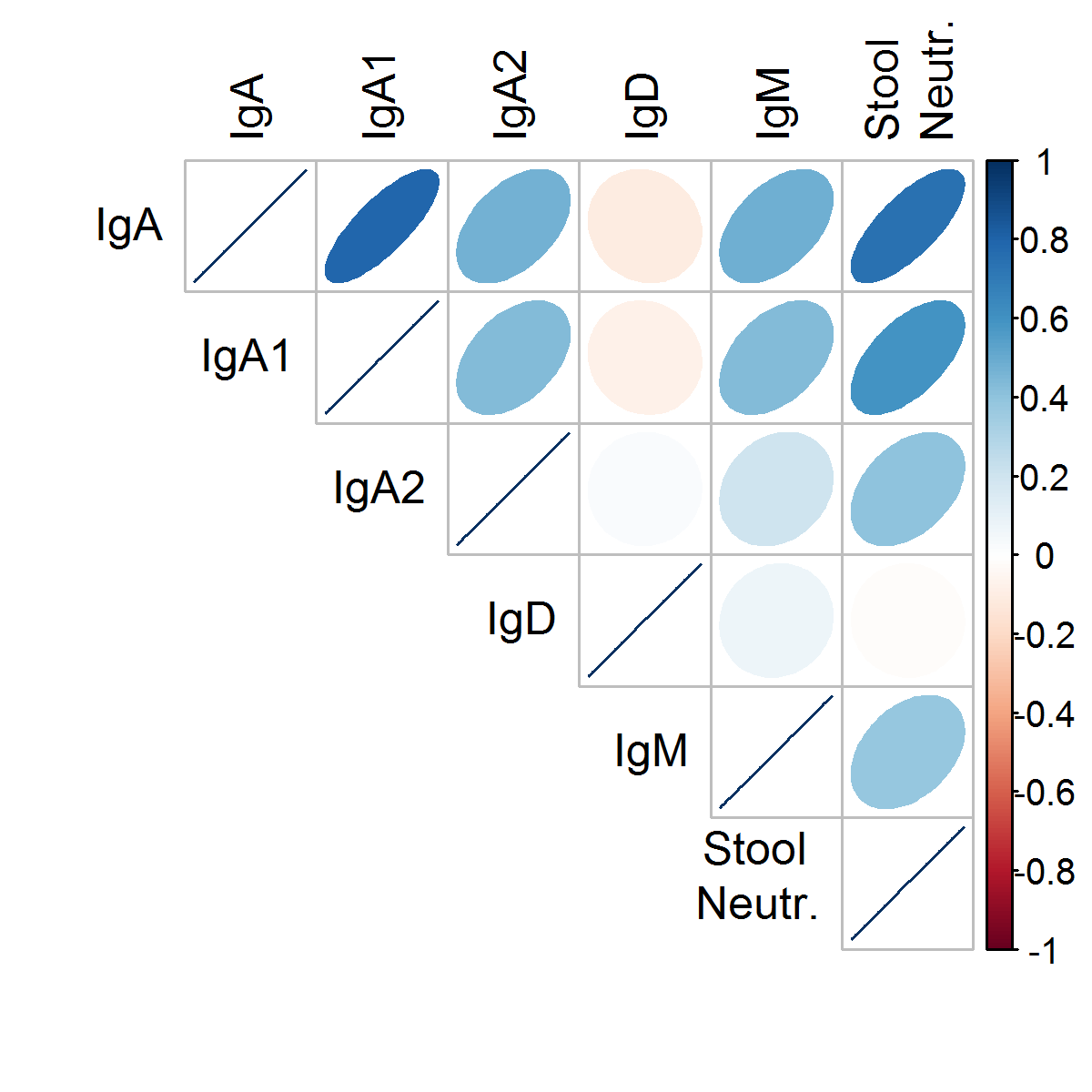 | 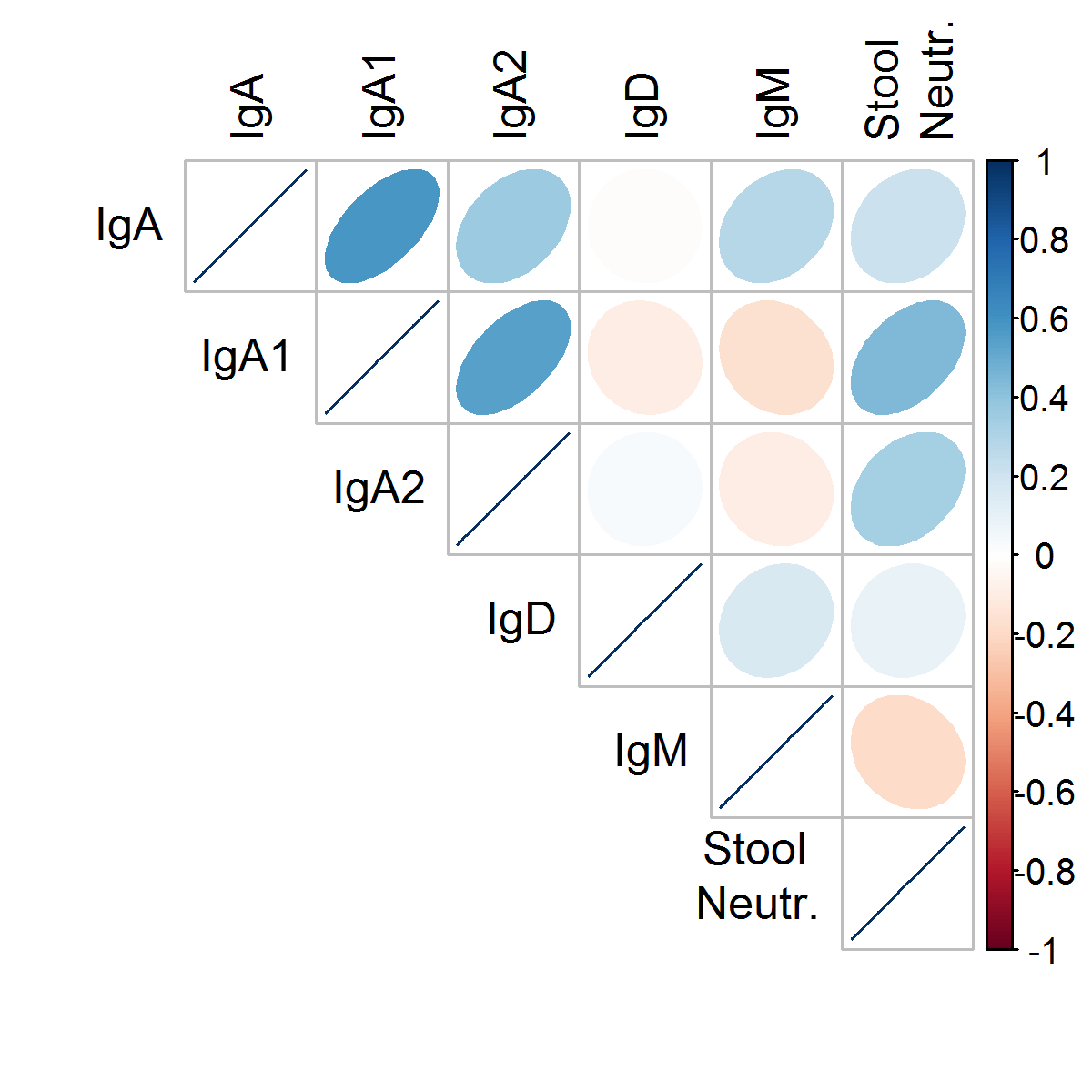 |
|  | **C. Three Weeks** | 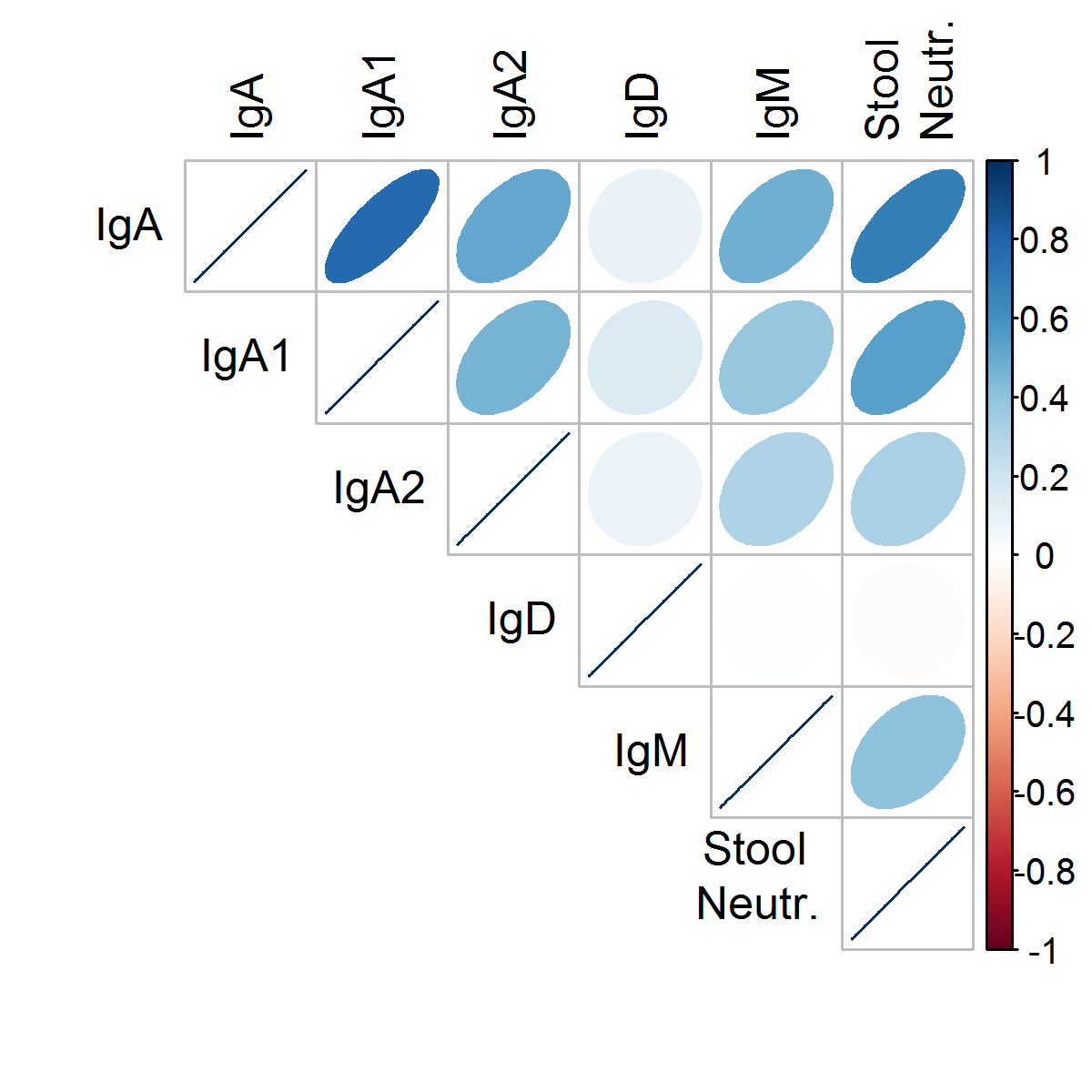 | 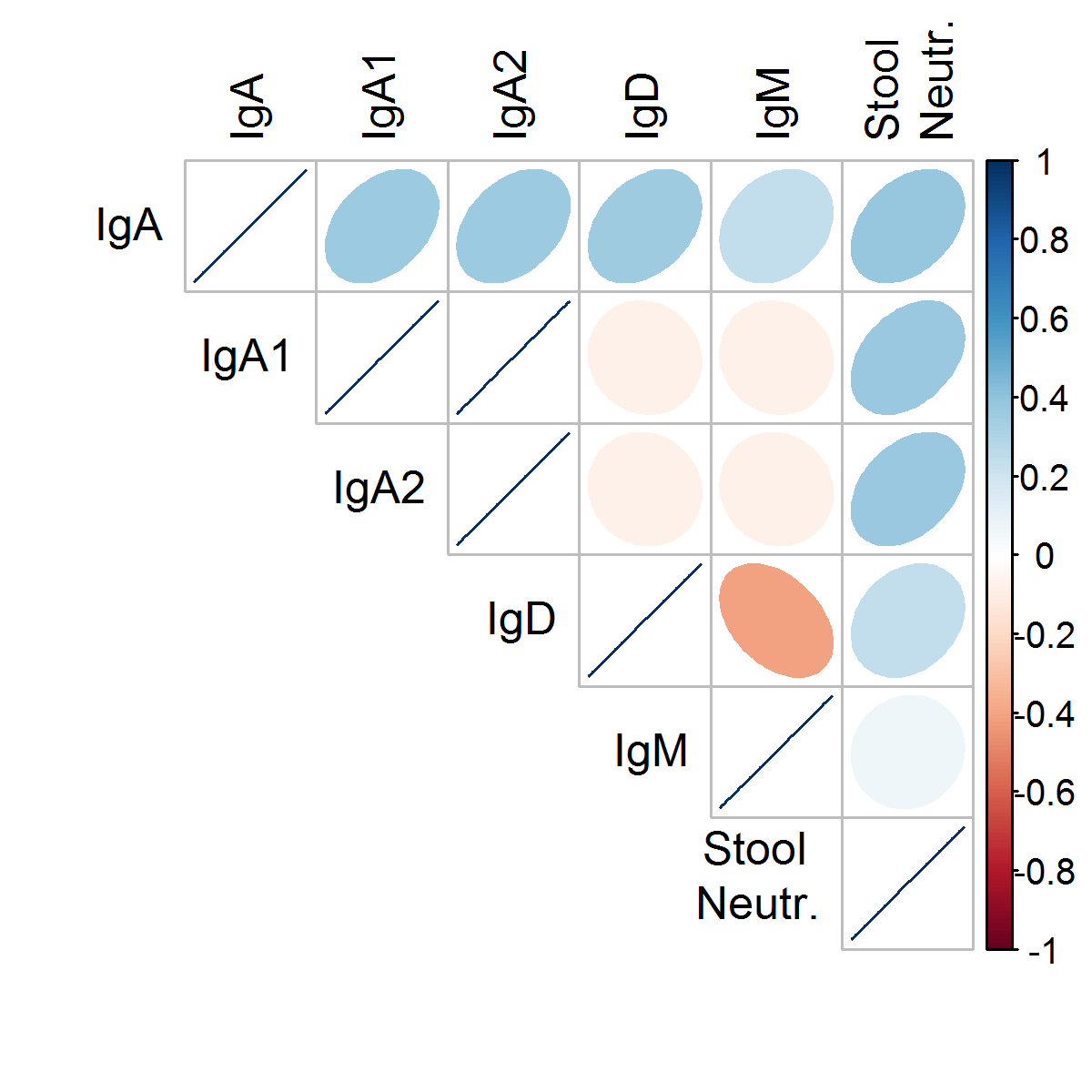 |

**Supplementary Figure 4. Correlation between viral shedding (CCID_50_) and polio type 2-specific neutralization titer measured in serum 1 week after challenge in infants with any viral shedding detectable during post-challenge study visits (N=79).**  Black squares and bars indicate the median and interquartile ranges of the variables on the y-axes within each quintile of serum neutralization plotted against the mean serum neutralization within each quintile. Red markers indicate infants in the bOPV-bOPV-bOPV+IPV group (N=37); gray markers indicate infants in the bOPV-bOPV-bOPV+mIPV2HD group (N=42). Abbreviations: IPV = trivalent inactivated polio vaccine; mIPV2HD = monovalent high dose inactivated polio type 2 vaccine; CCID_50_ = 50% cell-culture infectious dose; mOPV2 = monovalent oral polio vaccine type 2.
